# Neuropsychiatric symptoms in patients with dementia in primary care: a study protocol

**DOI:** 10.1186/1471-2318-14-32

**Published:** 2014-03-17

**Authors:** Petra Borsje, Roland B Wetzels, Peter LBJ Lucassen, Anne-Margriet Pot, Raymond TCM Koopmans

**Affiliations:** 1 Department of Primary and Community Care, Radboud university medical centre, PO Box 9101, 6500 HB Nijmegen, The Netherlands; 2 Thebe, Region South-East, Bergstraat 6, Goirle 5051 HC, The Netherlands; 3 Department of Clinical Psychology, Faculty of Psychology and Education, VU University Amsterdam, Van der Boechorststraat 1, Amsterdam 1081 BT, The Netherlands; 4 Trimbos Institute, Netherlands Institute of mental Health and Addiction, Da Costakade 45, Utrecht 3521 VS, The Netherlands

**Keywords:** Dementia, Neuropsychiatric symptoms, Primary care, Longitudinal, Observational, Quality of life, Caregiver distress

## Abstract

**Background:**

Neuropsychiatric symptoms (NPS) frequently occur in patients with dementia. To date, prospective studies on the course of NPS have been conducted in patients with dementia in clinical centers or psychiatric services. The primary goal of this study is to investigate the course of NPS in patients with dementia and caregiver distress in primary care. We also aim to detect determinants of both the course of NPS in patients with dementia and informal caregiver distress in primary care.

**Methods/design:**

This is a prospective observational study on the course of NPS in patients with dementia in primary care. Thirty-seven general practitioners (GPs) in 18 general practices were selected based on their interest in participating in this study. We will retrieve electronic medical files of patients with dementia from these general practices. Patients and caregivers will be followed for 18 months during the period January 2012 to December 2013. Patient characteristics will be collected at baseline. Time to death or institutionalization will be measured. Co-morbidity will be assessed using the Charlson index. Psychotropic drug use and primary and secondary outcome measures will be measured at 3 assessments, baseline, 9 and 18 months. The primary outcome measures are the Neuropsychiatric Inventory score for patients with dementia and the Sense of Competence score for informal caregivers. In addition to descriptive analyses frequency parameters will be computed. Univariate analysis will be performed to identify determinants of the course of NPS and informal caregiver distress. All determinants will then be tested in a multivariate regression analysis to determine their unique contribution to the course of NPS and caregiver distress.

**Discussion:**

The results of this study will provide data on the course of NPS, which is clinically important for prognosis. The data will help GPs and other professionals in planning follow-up visits and in the timing for offering psycho-education, psychosocial interventions and the provision of care. In addition, these data will enlarge health professionals’ awareness of NPS in their patients with dementia.

## Background

Dementia is a chronic and mostly progressive disease with great impact on patients and their family members. Current estimates indicate that 35.6 million people worldwide are living with dementia. This number is expected to double by 2030 and more than triple by 2050 [1]. The estimated standardized prevalence of dementia among persons aged 60 and over in Western Europe, Central Europe and Eastern Europe is 7.3%, 5.8% and 5.7%, respectively [1]. In the Netherlands 250,000 people have dementia, and most of these patients reside in the community.

Neuropsychiatric symptoms (NPS), such as psychosis (delusions and hallucinations), depressive mood, anxiety, irritability/lability, apathy, euphoria, disinhibition, agitation/aggression, aberrant motor activity, sleep disturbance and eating disorder, often occur in patients with dementia. NPS result in lower quality of life for both the patient and caregiver and affect the quality of the patient-caregiver relationship [2,3]. NPS, severity of cognitive impairment, Alzheimer’s dementia, high rates of functional dependence and depressive symptoms are predictors of nursing home admission [4]. Major depression is a predictor of early institutionalization in the first year following the dementia diagnosis [5]. The baseline severity of NPS, stage of dementia and use of support services predict the future severity of NPS [6]. However, there is a lack of knowledge about the determinants of neuropsychiatric symptoms in community-dwelling patients with dementia.

Studies in various countries reported NPS prevalence rates that ranged from 66 to 94% [7-13]. Incidence rates of NPS were reported in only a few studies. In a United States (US) Cache County study, Steinberg et al. found an incidence rate of 69% after an 18-month follow-up [14]. Only one Dutch study has been conducted on the course of NPS in community-dwelling people with dementia. In the MAASBED (MAAstricht Study of BEhaviour in Dementia) study it was found that 81% of the patients with dementia from a memory clinic and an ambulatory mental health institute showed any type of NPS, such as agitation (19%), irritability (24%), aberrant motor behavior (26%), depression (35%), apathy (40%), anxiety (21%) and delusions (22%) [15]. After a 6-12-month follow-up, the cumulative incidence of NPS was 74% [15]. Several prospective studies of NPS in community-dwelling patients with dementia have been conducted in other countries. In most of these studies, the Neuropsychiatric Inventory (NPI) was used to evaluate NPS. Eighty-one percent of those without any NPS at baseline had at least one symptom after 18 months in the US Cache County study [16]. In the same study, 67% of the participants with at least 1 clinically significant NPS (total NPI score ≥4) at baseline continued to display clinically significant NPS after 18 months. Among the 10 neuropsychiatric symptoms assessed at baseline, delusions persisted in 66%, depression in 58% and aberrant motor behavior in 56% of the individuals. Hallucinations and disinhibition persisted in 25% and 11% of the participants, respectively [16]. In a study in the United Kingdom, 94% of the participants had at least one NPS and 75% of the participants had an NPI score ≥4 for at least one symptom. Of the latter group, 80% had a persistent NPI score ≥4 in at least one domain after 6 months [12]. Furthermore, a 2-year follow-up study in England and Wales showed that NPS co-occur [17]. Anxiety and depression as well as misidentification, persecution and hallucinations were strongly associated [17]. The REAL-FR (Réseau sur la Maladie d’Alzheimer Français) cohort study found that the percentage of patients presenting one or more clinically relevant NPS as measured by the NPI increased from 66% at baseline to 88% after 4-year follow-up [13]. Prevalence of agitation increased from 17.9% to 29.1%, apathy from 43.0% to 62.9%, disinhibition from 2.6% to 14.6%, hallucination from 2% to 4.6% and aberrant motor behavior from 13.9% to 29.1%. Prevalence of hyperactivity and apathy increased significantly during the follow-up, whereas the prevalence of affective and psychotic symptoms did not increase [13].

To date, prospective studies on the course of NPS have been conducted on ambulatory patients with dementia in memory clinics or clinical centers [13,15], using ambulatory services [15] or who were approached through local psychiatric services, the volunteer sector and nursing and residential care homes [12]. High (cumulative) prevalence and (cumulative) incidence rates of NPS were found in these studies. In the REAL-FR cohort, a prevalence of 66% at baseline increased to 88% after 4 years [13]. In the MAASBED study, a prevalence of 80.9% at baseline increased to a cumulative prevalence of 88.9% after 2 years, and the cumulative incidence after 6–12 months was 74% in ambulatory patients of memory clinics or psychiatric services [15]. In the LASER-AD (London And the South East Region - Alzheimer’s Disease) study, 33% of the participants were recruited from 24-hour care settings and 67% were living at home. The prevalence rates of NPS at baseline were 93.8% for at least one NPS, and 88.4% of the participants had a NPI score ≥ 4 in at least one domain. At 6-month follow-up, 96.2% had at least one NPS in any domain. Of these participants 80.3% had a persistent NPI score ≥ 4 in at least one domain [12].

Steinberg and Savva have studied a community-dwelling population [16,17]. In Cache County, 62% of the participants with dementia had at least one NPS at baseline and 23% had a NPI score ≥ 4 in at least one domain. After 18 months, 95% of the participants had at least one NPS at baseline and 49% had a NPI score ≥ 4 in at least one domain. However, of the 5092 individuals who were enrolled in this study, 265 resided in nursing homes. Information was not provided on the percentage of the 329 participants with dementia who resided in nursing homes [16]. In the Medical Research Council Cognitive Function and Aging Study, prevalence rates of 5.8% for confabulation to 50.3% for apathy were found in dementia patients. Incidence rates of 2% for anxiety to 61% for apathy were found after 2 years. Furthermore, persistence rates were 13% for confabulation and 66% for apathy. The percentage of participants who lived in institutions was 38% at baseline and 66% after 2-year follow-up.

It appears as though the prevalence and incidence rates of NPS in community-dwelling patients with dementia are lower (23% to 50% and 49% to 60% respectively) than those of ambulatory patients of memory clinics or clinical centers and ambulatory patients of psychiatric services (66% to 96.2% and 74% respectively). Finally, the relationship between caregiver characteristics and caregiver distress and NPS was unclear in these studies.

### Aims of the study

The first aim of this study is to investigate the course of NPS in patients with dementia and informal caregiver distress in primary care. We also aim to detect determinants of both the course of NPS in patients with dementia and informal caregiver distress in primary care.

## Methods/design

### Study design

This is a prospective observational cohort study in primary care. For this study, all 192 known general practitioners (GPs) in 114 general practices in the region West- and Middle-Brabant in the southern part of the Netherlands were invited to participate. All GPs of the 114 practices individually received a letter with information on the study and were invited to attend a meeting about NPS and the study. Announcements of this study were also posted on the websites of the regional GP corporations. Thirty-seven GPs in 18 general practices were selected based on their interest in participating in the study. The presence of specialized care for elderly people in the general practices will be determined by asking whether the participating GPs followed a specialized management training course in elderly care medicine in primary care and whether specialized staff members are available in these general practices to support the GP in managing the care for elderly patients. We will retrieve electronic medical files of patients with dementia from these general practices. Patients and informal caregivers will be approached by letter. Informal caregivers are persons who are listed in the electronic medical files of the GP as the main informal caregiver and contact person. There will be no restriction in the amount of time that the informal caregiver spends with the patient. After the letter is mailed to the patient and informal caregiver, the GP will contact the patient or informal caregiver by telephone to stimulate participation in the study. The assessment interviews will take place at the patients home by a trained interviewer.

Patients and informal caregivers will be followed for 18 months. In case that a patient dies or will be institutionalized, length of time to death or institutionalization, respectively, will be measured. The study began in January 2012 and will end in December 2013.

In the Netherlands, many psychosocial interventions and care services are available for community-dwelling people with dementia, including cognitive training and stimulation, physical exercise, reminiscence, education and support for both patient and informal caregiver and respite care. Dementia CM is stimulated by the Dutch government and is available in all parts of the country. Dementia CM involves assessment, planning and advocacy for patients with dementia and their informal caregivers. It also aims to empower informal caregivers and facilitate timely access to essential care services to support their caregiver needs. In the southern region of the Netherlands, dementia CM is provided by many care organizations and care services. We consider this single component dementia CM.

In 14 of the participating general practices, a multicomponent collaborative care program named CONCERN (Care Optimization for Non-professional Caregivers of Elderly with dementia and Reduction of Neuropsychiatric symptoms) will be provided. In CONCERN, a dementia CM together with an elderly care physician (ECP) and the GP focus on optimization of care and improvement of quality of life for patients with dementia suffering from NPS and their informal caregivers. Following assessment and diagnosis of the NPS, a care plan is designed for the treatment and support of both the patient and informal caregiver. This care plan is periodically evaluated in a multidisciplinary meeting with the GP, ECP, dementia CM and other involved care services.

We will measure whether the patients with dementia and their informal caregivers are treated by single component dementia CM, CONCERN or care as usual (no CM).

### Patients and their informal caregivers

All patients in the participating general practices with a diagnosis of dementia as registered in the electronic medical files of the general practice, and living at home are eligible to participate in this study together with their informal caregiver (spouse, child or neighbor). We will select patients with the International Classification of Primary Care (ICPC) code for dementia (P70) from the electronic medical systems. This code includes Alzheimer’s disease and senile dementia. We will also select patients with memory disturbance (ICPC code P20) who are diagnosed with dementia. Patients with an estimated life expectancy of less than 3 months will be excluded from the current study. All patients and caregivers will receive a complete written description of the study and be asked to sign an informed consent document. If the patient is unable to provide informed consent, his or her legal representative will be asked to provide informed consent on the patient’s behalf.

### Ethical approval

This research project was presented for medical ethics review at the Committee on Research Involving Human Subjects (CMO) of the district Arnhem - Nijmegen, the Netherlands. The CMO judged that the current project is not subject to the Medical Research Involving Human Subjects Act (WMO) and can be conducted without review by the CMO.

### Assessment instruments

Data are collected by a trained research assistant during an interview with the patient and the caregiver at home at baseline (T0), after 9 months (T1) and at 18 months (T2). The same set of questionnaires will be used in all 3 assessments (Table 1). The outcome measures have good psychometric properties. The primary outcome for the patient is the NPI and that for the informal caregiver is the sense of competence (SCQ).

**Table 1:** Assessment instruments

	**Instrument**	**T0**	**T1**	**T2**
	Baseline variables	X		
**Patient**	Mini Mental State Examination	X	X	X
	Neuropsychiatric Inventory	X	X	X
	Cohen-Mansfield Agitation Inventory	X	X	X
	Cornell Scale for Depression in Dementia	X	X	X
	Quality of life in Alzheimer’s disease	X	X	X
	Charlson Index	X		
	Psychotropic drug use	X	X	X
**Informal caregiver**	Center for Epidemiological Studies Depression Scale	X	X	X
	General Health Questionnaire	X	X	X
	EuroQol	X	X	X
	Sense of Competence	X	X	X

### Patient characteristics

The following patient characteristics will be collected at baseline (T0): age, gender, marital state, socio-economic status/educational level and profession, use of health care services (psychiatric services; home care: nursing, domestic; day care services; on waiting list for residential care facility or nursing home). Co-morbidity will be assessed using the Charlson index (CI). The CI comprises 19 categories of International Classification of Diseases, 9th Revision, Clinical Modification (ICD-9CM) diagnose codes and is based on a set of risk factors for 1-year mortality risk [18]. The CI contains a weighted index for each disease, with a score that is a significant predictor of 1-year survival. Psychotropic drug use (antipsychotics, anticonvulsants, antidepressants, anxiolytics, hypnotics and medication for dementia) will be collected in all 3 assessments.

#### Neuropsychiatric symptoms

The Neuropsychiatric Inventory (NPI), developed by Cummings [19,20], will be the primary outcome. This inventory assesses 12 neuropsychiatric symptoms in dementia outpatients. The validity and reliability of the NPI [21] and of its Dutch version [22] were previously established. Since then, the NPI has been the most widely used rating scale for the assessment of NPS. The NPI comprises 12 categories of problem behavior, as follows: delusions, hallucinations, agitation/aggression, depression, anxiety, euphoria, disinhibition, irritability/lability, apathy, aberrant motor activity, sleeping disorder and eating disorder. For each positive symptom, the severity and frequency are scored on the basis of structured questions administered to the patients’ caregiver. The continuous score for each symptom is obtained by multiplying severity (1–3) by frequency (1–4). In line with previous studies [8,13-16], a score of 4 or more on one symptom will be taken to indicate the presence of specific ‘clinically relevant’ symptoms. Caregiver distress is also assessed (0–5), but is not calculated in the NPI total score. Frequency and severity scores of individual symptoms can be multiplied (FxS score) and summed over 12 items, yielding a total NPI score that ranges from 0 to 144. The following five NPI factor scores (based on the findings of previous studies) will be used [23,24]: (1) agitation, consisting of agitation/aggression, euphoria, disinhibition and irritability; (2) depression, consisting of depression and anxiety; (3) psychosis, consisting of hallucinations and delusions; (4) psychomotor agitation, consisting of aberrant motor behavior and nighttime behavior, and (5) apathy, consisting of apathy and eating disorder [23]. The NPI will be assessed by a trained interviewer during an interview with the informal caregiver.

The Cohen-Mansfield Agitation Inventory (CMAI), originally developed by Cohen-Mansfield [25], is the most widely used assessment scale for measuring the frequency of agitation and aggression. This inventory defines agitation as inappropriate verbal, vocal or motor activities not explained by apparent needs or confusion. The informant is the patient’s caregiver. Symptoms are assessed for the preceding 2 weeks. The original and translated Dutch version were found to be valid and reliable [26-28]. It consists of 29 individual items and can be categorized in 3 subscales, which assess physically aggressive (directed against a person or object), physically non-aggressive (not directed against a person or object, such as pacing and wandering) and verbally agitated behavior. Items are scored on a 7-point frequency scale, as follows: 1 = never; 2 = < once a week; 3 = 1–2 times per week; 4 = several times per week; 5 = 1–2 times per day; 6 = several times per day; 7 = several times per hour [26]. In community-dwelling persons with Alzheimer’s disease, the CMAI appears useful as an overall measure of behavioral disturbances, but scoring by subscale does not seem applicable [29].

The Cornell scale for depression in dementia (CSDD) is widely used for the screening of depressive symptoms in dementia. The CSDD consists of 19 items, each rated as 0 = absent, 1 = mild or intermittent or 2 = severe. The scores of the individual items are summed, and a cut-off of 8 or more indicates depression [30]. With a cut-off value of > or = 6 the CSDD has a sensitivity and specificity of 93% and 97%, respectively. It seems equally valid in demented and non-demented populations [31]. The CSDD will be administered by interviewing the informal caregivers about their observations of the patients’ behavior.

#### Cognition

Cognition will be assessed by the Mini-Mental State Examination (MMSE), which is the most widely used screening instrument to detect cognitive impairment [32]. It has a fair reliability and construct validity, with a high sensitivity for moderately to severe cognitive impairment and a lower sensitivity for mild cognitive impairment [33]. It comprises items that test orientation, attention, memory, language and constructive abilities. An important bias in using the MMSE is the extensive use of language, which leads to unreliable results in aphasic patients and patients who are incapable of speaking the local language [33].

#### Quality of life

The Quality of life in Alzheimer’s disease (Qol-AD) is used to measure quality of life. It is an easy-to-use 13-item instrument that covers physical health, energy, mood, living situation, memory, family, marriage, friends, self as a whole, ability to do chores around the house, ability to do things for fun, money and life as a whole. Each of the 13 items is rated on a 4-point Likert scale, as follows: 1 - ‘poor’; 2 - ‘fair’; 3 - ‘good’ and 4 - ‘excellent’ [34,35]. Logsdon found satisfactory validity and reliability, but a limited use for patients with an MMSE score of less than 10 [36]. In other studies, the Qol-AD showed very good psychometric properties, with satisfactory reliability and validity. Furthermore it can be completed with people with a wide range of severity of dementia [37-40].

### Informal caregiver characteristics

The following general characteristics of the informal caregivers will be collected at baseline (T0): age, gender, marital state, socio-economic status/educational level and profession.

#### Impact on informal caregiver

The psychological burden of caring for a patient with dementia, measured using the sense of competence (SCQ), will be the primary outcome for the informal caregivers. The SCQ is based on the family-crisis model [41] and derived from Zarit’s Burden Interview [42]. This interview was developed for informal caregivers of patients diagnosed with dementia and consists of 27-items that are rated on a 5-point scale, as follows: 1 ‘yes, completely agrees’ , 2 ‘yes, agrees’ , 3 ‘on the one hand agrees but on the other hand disagrees’ , 4 ‘no, disagrees’ and 5 ‘no, completely disagrees’ [43,44]. The SCQ consists of the following three subscales: 1. satisfaction with the elderly person as the recipient of care (7 items; range 7–35; Cronbach’s alpha = 0.55); 2. satisfaction with one’s own performance as a caregiver (12 items; range 12–60; Cronbach’s alpha = 0.63); and 3. consequences of involvement in care for the personal life of the caregiver (8 items; range 8–40; Cronbach’s alpha = 0.50). For each dimension, higher scores indicate a better sense of competence. Overall sum-scores range from 27 to 135 [43,45,46]. The validity and usefulness of the SCQ when applied to informal caregivers of older adults with dementia symptoms (i.e., cognitive impairment, pre-diagnostic dementia or dementia in its early stages) has also been studied. The 3 subscales of the SCQ showed good homogeneity and feasibility, but their construct validity was insufficient. Only the subscale ‘consequences of involvement in care for the personal life of the caregiver’ was found to be partly valid [44].

#### Depression

The Center for Epidemiological Studies Depression Scale (CES-D) is a 20-item instrument that assesses the frequency of experienced depressive symptoms within the past week. The items are rated on a 4-point Likert scale from 0 ‘rarely or none of the time to 3 ‘most or all of the time’. Scores range from 0 to 60. A score of 16 or over has been clinically associated with a greater risk of depression [47,48]. Test-retest reliability at 3-month intervals over a 12-month period for the CES-D was reported to be 0.49–0.54 [47]. This instrument has been widely used in dementia research and most of these studies have used the CES-D total score [49-54]. The original 4-factor model of item responses is informative for identifying meaningful clusters of depressive symptoms in dementia caregivers [55,56].

#### General health

The General Health Questionnaire (GHQ-12) is a 12-item questionnaire, with sum scores ranging from 0 to 36 (lower scores indicate better health status) [57]. It is a widely used self-report instrument, that is assumed to cover a wide range of common psychiatric morbidity, in particular, anxiety and depressive disorders. The GHQ was originally developed as a screening instrument for use in general practice. Several short-form versions (30, 28, 20 and 12 items) of the original 60-item version have been developed. Good psychometric properties have been reported, in particular for the GHQ-12 [58,59].

EuroQol (EQ-5D) is a self-administered questionnaire in which respondents evaluate their health state “today” on the following 5 dimensions: mobility, self-care, usual activities, pain/discomfort and anxiety/depression. A 1-to-3 scale is used for each dimension, representing no problem, some problem, or extreme problem for the subject to engage in the activity alone; for the pain and anxiety items, the three ratings relate to the severity of symptoms. The instrument also has a visual analog scale “thermometer” (VAS), a 20-cm scale anchored at 0 “worse imaginable health state” and 100 “best imaginable health state” [60]. The EQ-5D has been translated into several languages and has been validated and employed in many studies on general populations and subjects with mild dementia [61,62].

### Data analysis

All data will be analyzed using the Statistical Package for Social Science 20.0 (SPSS 20.0). Descriptive analysis will be used for general patient and caregiver characteristics, disease characteristics and time to death or time to institutionalization. Only data of patients and caregivers with complete follow-up of 18 months will be used for data analysis. Patient and caregiver characteristics of withdrawals (subjects included, but no data received) and losses to follow-up/drop-outs will be described and compared with the patients and caregivers who will complete follow-up. If patients become institutionalized during follow-up, data collection will be continued with the same informant/informal caregiver. Patient and caregiver characteristics, baseline MMSE and baseline NPI total scores will be compared to the non-institutionalized subjects. If these data are comparable, then they will be used for data analysis.

The frequency (point and cumulative prevalence), cumulative incidence, and persistence of symptoms are expressed as the percentage of patients with scores greater than 3 on any item of the NPI, at study onset and/or at any follow-up evaluations. Point prevalence will be defined as the proportion of patients with specific symptoms at each assessment. The accumulative prevalence will be defined as the proportion of patients developing a specific symptom on at least one assessment over the 18-month study period. The cumulative incidence will be rated as the proportion of patients who are symptom-free at baseline but develop the specific symptom at subsequent assessments. A symptom will be considered as persistent if it was present on at least two subsequent assessments, regardless of time of first manifestation of the symptom. In addition, the proportion of patients with persistence of symptoms during all 3 assessments will be calculated.

Univariate analysis will be performed to identify determinants of NPS in patients with dementia in primary care as dependent variable for each assessment. Univariate analysis will also be performed to identify determinants of caregiver distress as dependent variable. Independent determinants will be multicomponent collaborative care (CONCERN), single component dementia CM, NPS at baseline, cognition and use of health care services (home care: nursing and domestic; use of day care services).

All determinants will then be tested in a multivariate regression analysis to determine their unique contribution to the course of NPS and informal caregiver distress. To take into account the clustering of patients with dementia/informal caregivers in general practices and the repeated measurements within patients random coefficient analyses will be used.

According to the National Public Health Compass, developed and coordinated at the Dutch National Institute of Public Health and the Environment, absolute prevalence of patients with dementia in registrations of general practices is 20 per general practice per year. Based on their interest in participating 18 practices were selected. With an assumed response rate of 50% and loss to follow-up rate of 30% after 18 months, the expected study population will be 126 patients with dementia. In analysis of causal influences in observational data, as a rule of thumb 1 candidate predictor can be studied for every 10 patients. For logistic regression this rule can be relaxed to 5–9 events per candidate predictor [63]. The assumed prevalence rate of NPS in primary care is 60% [16,17]. The number of independent variables in this study will be 7. Therefore 126 patients with dementia will suffice for the regression analyses.

Proportions (prevalence, incidence, persistence) can be estimated with absolute precision of 10% and a confidence level of 95% taking into account design effect of 1.25 based on an intraclass correlation coefficient (ICC) of 0.05 and a mean cluster size of 6, assuming a conservative estimate of anticipated proportion of 50%.

## Discussion

To our knowledge, this study is the first to focus on the course of NPS in patients with dementia and informal caregiver distress in primary care. All selected outcome measures have been proven and validated. The data will be collected by one research assistant. Therefore, measurement inaccuracies will be minimal.

This study has some limitations. Only 19% of the GPs we invited are willing to participate. This might limit the generalizability of the findings. Data will be collected at baseline, after 9 months and after 18 months. Variations in course between two successive assessments will be unknown. Because this is a naturalistic study, the course of NPS can be influenced by psychosocial and pharmaceutical interventions that we will not specifically assess in this study. Furthermore, we will select patients coded with dementia as classified in the ICPC code P70 and P20. Dementia in these patients is not necessarily defined with international criteria and Dutch consensus guidelines, causing a risk of bias. In the different general practices variability exists in the usage of the classification according to ICPC in the electronic medical files. However, because GPs often wait before diagnosing dementia, we expect that this bias will be small. On the other hand, this may bias the sample towards a more severe spectrum of illness.

The current study will provide more detailed information about consequences of NPS for the quality of life of both patients and informal caregivers as well as the influence of NPS on depressive symptoms and experienced health state of the caregiver, which is clinically important. The data will help GPs and other professionals in planning follow-up visits and in the timing of offering psycho-education, psychosocial interventions and the provision of care. It will enlarge their awareness of NPS in their patients with dementia. An individually tailored approach for patients with dementia and their informal caregivers may offer more and better treatment opportunities.

## Abbreviations

CES-D: Center for Epidemiological Studies Depression Scale; CI: Charlson index; CM: Case manager; CMAI: Cohen-Mansfield Agitation Inventory; CMO: Committee on Research Involving Human Subjects; CONCERN: Care optimization for Non-professional Caregivers of Elderly with dementia and Reduction of Neuropsychiatric symptoms; CSDD: Cornell Scale for Depression in Dementia; ECP: Elderly care physician; EQ-5D: EuroQol; GHQ: General Health Questionnaire; GP: General practitioner; ICC: Intraclass correlation coefficient; ICD-9CM: International Classification of Diseases, 9th revision, Clinical Modification; ICPC: International Classification of Primary Care; LASER-AD: London And the South East Region – Alzheimer’s Disease; MAASBED: MAAstricht Study of Behaviour in Dementia; MMSE: Mini-Mental State Examination; NPI: Neuropsychiatric Inventory; NPS: Neuropsychiatric symptoms; Qol-AD: Quality of life in Alzheimer’s Disease; REAL FR: Réseau sur la Maladie d’Alzheimer Français; SCQ: Sense of competence; SPSS 20.0: Statistical Package for Social Science 20.0; US: United States; VAS: Visual analog scale; WMO: Medical Research Involving Human Subjects Act.

## Competing interests

The authors declare that they have no competing interests.

## Authors’ contributions

PB is the primary investigator of this study, she designed the study and wrote the manuscript. The collected data will be processed and analyzed by PB. RW and RK participated in the design of the study, reviewed this study protocol and will help in the analysis of the data. PL and AP participated in the design of the study and reviewed this study protocol. All authors have given final approval of the version to be published.

## Pre-publication history

The pre-publication history for this paper can be accessed here:

http://www.biomedcentral.com/1471-2318/14/32/prepub
